# Embolization of the false lumen using IMPEDE-FX embolization plugs as part of treatment of an infrarenal post-dissection aneurysm: a case report

**DOI:** 10.1186/s42155-020-00183-6

**Published:** 2020-12-04

**Authors:** Anne-Jet S. Jansen, Paul M. van Schaik, Jasper M. Martens, Michel M. P. J. Reijnen

**Affiliations:** 1 Department of Surgery, Rijnstate, P.O. Box 9555, 6800 TA Arnhem, The Netherlands; 2 Department of Radiology, Rijnstate, Arnhem, The Netherlands; 3 Multi-Modality Medical Imaging Group, TechMed Centre, University of Twente, Enschede, The Netherlands

**Keywords:** Embolization, Therapeutic, Post-dissection aneurysm, False lumen, SMP plug, IMPEDE-FX, Embolization plug, Infrarenal

## Abstract

**Background:**

This case report demonstrates the value of IMPEDE-FX plugs in an embolization procedure of a false lumen of an infrarenal post-dissection aneurysm.

**Case presentation:**

A 69-year-old patient was treated with mitral valve replacement, complicated by a Stanford type-A dissection. After 9 years he presented with an enlarging infrarenal post-dissection aneurysm. The false lumen was embolized using multiple IMPEDE-FX plugs as part of the treatment in addition to embolization of the inferior mesenteric artery and overstenting of the re-entry in the right iliac artery. At 15 months the CTA showed a fully thrombosed false lumen and remodeling of the true lumen.

**Conclusions:**

The false lumen of an infrarenal post-dissection aneurysm can successfully be embolized using IMPEDE-FX embolization plugs as part of the treatment strategy. Prospective trials on patients with non-thrombosed false lumina are indicated.

## Background

Persistent false lumen filling is associated with failing remodeling of post-dissection aorta, resulting in an increased risk of aneurysm formation, rupture and death (Rohlffs et al. 2018; Song et al. 2007). Endovascular strategies aimed at promoting full thrombosis of the false lumen mostly focus on covering the entry-tear (Rohlffs et al. 2018; Song et al. 2007). If unsuccessful, embolization of the false lumen could be considered. Several techniques have been described, such as the use of conventional coils, plugs, glue, and iliac limb occluders (Hofferberth et al. 2012; Idrees et al. 2014), which are usually not suitable for large false lumen diameters. Kolbel et al. (Kölbel et al. 2013) developed the Candy-Plug (Cook Medical, Bjæverskov, Denmark) for the occlusion of a large false lumina, but this is not CE approved to date.

**Fig. 1:**
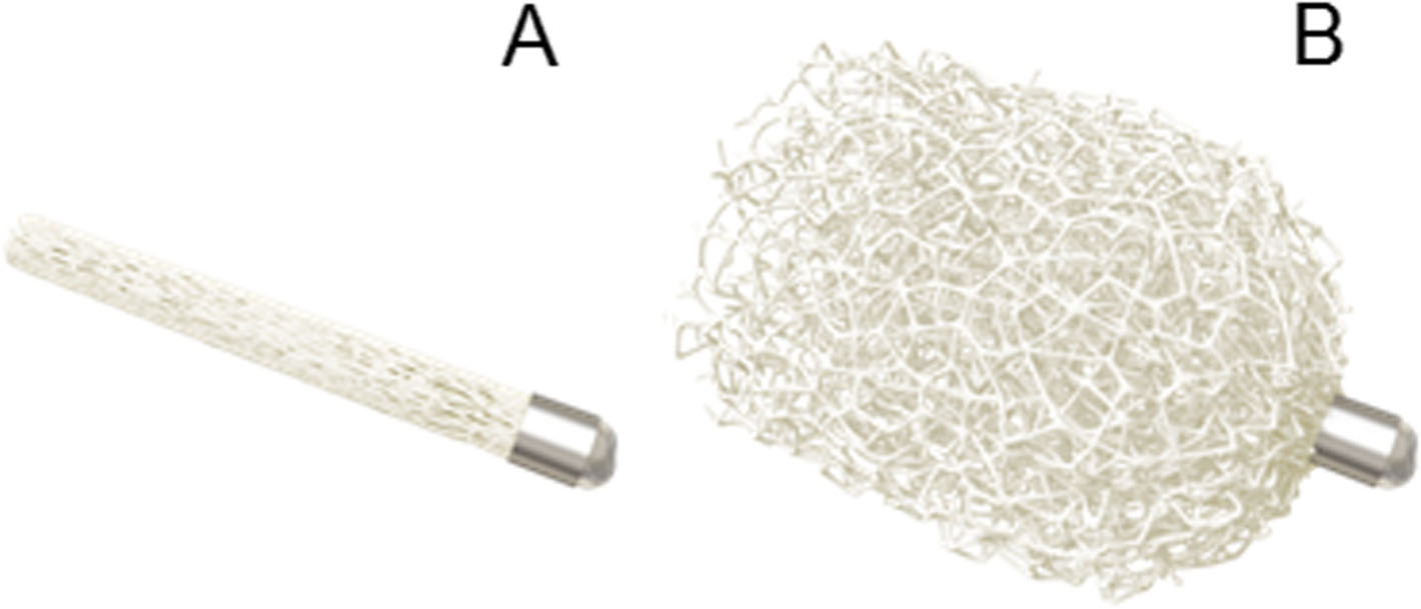
The IMPEDE-FX Embolization Plug in its crimped (**a**) and expanded (**b**) state. Images were obtained with the permission from Shape Memory Medical

The IMPEDE-FX Embolization Plug (Shape Memory Medical, Santa Clara, CA) is a novel embolization device consisting of a self-expanding Shape Memory Polymer (SMP) Plug and a marker band (IMPEDE-FX Embolization Plug Instructions for Use 2020). SMP is a porous, biocompatible and non-inflammatory polymeric scaffold, that has the ability to self-expand from its crimped state into its memorized shape by exposure to an aqueous environment and body temperature (Fig. 1a and b). SMP has been proposed as a suitable biomaterial for embolization applications due to their capability of shape recovery (Rodriguez et al. 2014a) and the interconnected, large surface area porosity (Singhal et al. 2012). This interconnected porosity serves as a scaffold for blood flow, thrombus formation, and tissue healing by supporting rapid formation of small interconnected clots throughout its structure. SMP provides a stable occlusion by promoting fast conversion to organized thrombus, followed by collagen deposition without chronic active inflammation. The SMP Plug offers a high embolic material volume and inherent 100% packing density (IMPEDE-FX Embolization Plug Instructions for Use 2020), important to avoid recanalization (Yasumoto et al. 2013). The IMPEDE-FX embolization plug is indicated to obstruct or reduce the rate of blood flow in the peripheral vasculature, and received CE Mark approval in 2018 in Europe.

In this report, we describe a case of an infrarenal post-dissection aneurysm that was successfully embolized using multiple IMPEDE-FX embolization plugs. Written informed consent was obtained from the patient for publication of this case report and any accompanying images.

## Case presentation

In 2010, a 69-year-old patient was treated in another hospital with a mitral valve replacement, complicated by a Stanford type-A dissection. In January 2019 he presented with an enlarging infrarenal post-dissection aneurysm with a maximum diameter of 81 mm. In 2010 this diameter was 52 mm.

**Fig. 2:**
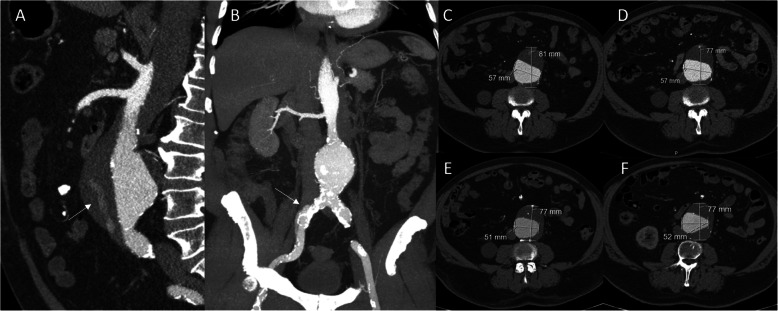
Preoperative CT angiography of the post-dissection infrarenal aneurysm in the sagittal plane showing the filling of the false lumen (arrow) (**a**), coronal maximum intension projection (MIP) reconstruction (**b**) and transversal slides of a CT angiography of the post-dissection infrarenal aneurysm before treatment (**c**), 1 month postoperatively (**d**), 8 months (**e**) and 15 months (**f**) postoperatively

Contrast-enhanced computed tomography (CTA) scanning showed a post-dissection infrarenal aneurysm without filling through the proximal entry tear. The original dissection originated from the aortic arch to the abdominal aorta and extended to the right common iliac artery (CIA) (Fig. 2a and b). Diameters at the thoracic level were below the threshold for intervention. The celiac trunk and superior mesenteric artery derived from the true lumen, while the left renal artery derived from the false lumen and was occluded. There was filling of the false lumen of the aneurysm through the inferior mesenteric artery (IMA) and the right external iliac artery (Fig. 2a). The maximum diameter of the aneurysm was 81 mm, with a true lumen diameter of 57 mm (Fig. 2c).

**Fig. 3:**
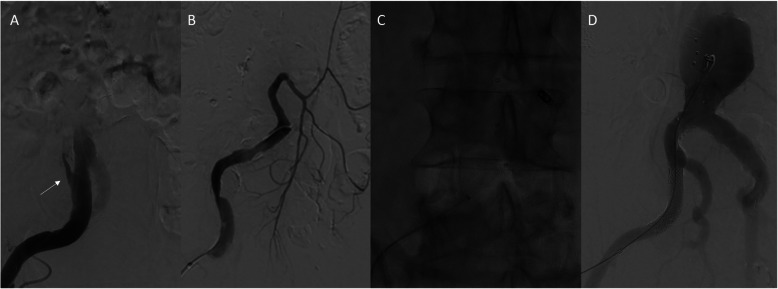
Procedural angiography showing the filling right iliac artery through the iliac entry tear (**a**), the filling of the false lumen and the IMA (**b**), the position of the SMP plugs (**c**), and completion angiography showing filling of the true lumen with complete obliteration of the false lumen (**d**)

After ample consideration and informed consent, patient was scheduled for embolization of the IMA and filling of the false lumen, using IMPEDE-FX embolization plugs. Patient was operated under general anesthesia and antibiotic prophylaxis. After placement of a 5-F sheath a blowback angiography was performed (Fig. 3a). Subsequently, the false lumen was cannulated. Angiography showed the false lumen and the IMA (Fig. 3b). Subsequently a 5-F sheath was advanced into the IMA, and it was embolized using a 5 × 80 mm Interlock-18 microcoil (Boston Scientific, Marlborough, MA) to prevent distal migration of the IMPEDE-FX Plug, just before its first bifurcation and proximal of Riolan, to guarantee collateral flow. Then an IMPEDE-FX-12 Embolization Plug was placed in the orifice of the IMA followed by 6 other IMPEDE-FX-12 Embolization Plugs, that filled the entire false lumen (Fig. 3d).

Afterwards, the true lumen was cannulated and, in order to cover the entry in the iliac artery, a 11mmx39 balloon-expandable covered stent (GORE®VIABAHN®VBX Balloon Expandable Endoprosthesis**,** W.L.Gore and associates, Flagstaff, AZ) was placed in external iliac artery. Completion angiography showed a fully excluded false lumen with patent flow through the true lumen. The most distal plug, however, seemed to have moved distally, indicating that the CIA entry was not fully covered. Therefore, the balloon-expandable covered stent was extended distally to the level of the deep inferior epigastric artery with a good result. The access site was closed using an Angio-Seal (Terumo Interventional Systems, Somerset, NJ) vascular closure device. The post-procedural course was uneventful and patient was discharged at the first postoperative day.

At one-month a CTA showed a fully thrombosed false lumen. The diameter of the aneurysm was 77 mm (Fig. 2d). At 8 and 15 months another CTA confirmed complete and persistent thrombosis of the false lumen. The total diameter of the false lumen remained stable (Fig. 2e and f). Furthermore, remodeling of the true lumen was evident. The shape of the true lumen had changed as the thrombus load at the left lateral side was increased (Fig. 2). The maximum diameter of the true lumen decreased from 56 mm to 52 mm. The volume of the true lumen showed a similar decrease, with a preoperative volume of 102.4 cm^3^, and a volume of 109.2 cm^3^ and 87.5 cm^3^ at 1 and 8 months, respectively.

## Conclusions

This case demonstrates the successful use of IMPEDE-FX embolization Plugs to obliterate a false lumen of an infrarenal post-dissection aneurysm. This method could provide an alternative technique for endovascular embolization of a larger false lumen in patients with an aortic dissection, also in the thoracic area. The ease of use, the option to use multiple plugs, controlled release and the off-the-shelve availability might facilitate treatment of a wide range of patients. The device also holds promise for patients with a persistent type II endoleak after EVAR. The polymer plugs cause only minimal radiographic artifacts and has a favorable property of rapid clot maturation (Landsman et al. 2016).

When using conventional coils a recanalization rate up to 20% has been described (Enriquez et al. 2013). They only induce fresh thrombus formation, whereas the IMPEDE-FX Plug provides a scaffold for tissue ingrowth. The plugs thus aim to minimize time to thrombus maturation by promoting initial clotting of blood within the scaffold, which will be replaced by connective tissue and this could reduce the risk of recanalization. In a porcine model, Rodriquez et al. (Rodriguez et al. 2014a; Rodriguez et al. 2014b) showed significant connective tissue infiltration throughout implanted SMP foams, causing complete and stable occlusion of treated intracranial aneurysms. The material is less inflammatory compared to traditionally used materials (Rodriguez et al. 2014a). Another advantage of the IMPEDE-FX embolization Plugs is slow degradation of the plugs. Most of the material is degraded at 180 days after implantation (FDA Report IMPEDE Embolization Plug, 5mm, IMPEDE Embolization Plug, 7mm, IMPEDE Embolization Plug, 10mm 2020).

**Fig. 4:**
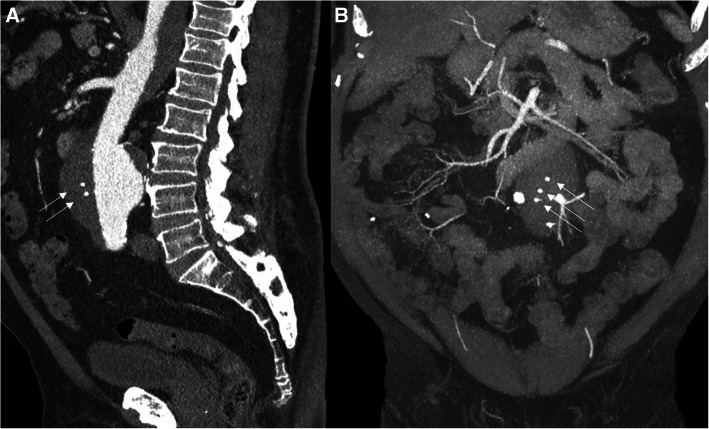
Maximum intension projection (MIP) reconstructions in the sagittal (**a**) and coronal (**b**) plane of the first post-procedural CT angiography showing markers of the plugs (arrows)

The plug displacement that occurred during the intervention was likely caused by the diameter mismatch of the plug and the lumen diameter. Larger plugs could have prevented this, but do not exist to date. Another option could have been the use of the IMPEDE, that does have a coil serving as an anchor, which could help with stabilization of the plug. One of the advantages of using the IMPEDE-FX is the lack of metal artifact on follow-up CT as demonstrated in Fig. 4.

In the current patient we have decided to leave the true lumen untreated, as both the diameter and volume decreased postoperatively. Endovascular treatment was considered to be challenging due to an extreme elliptical shape of the infrarenal neck, while open repair was considered to be very high-risk. The shape of the true lumen also changed and the volume decreased by about 15%, after a minor increase, which may be explained by the decrease of pressure in the false lumen.

In conclusion, this case suggest that IMPEDE-FX embolization plugs can be used successfully to embolize a false lumen of a post-dissection aneurysm, also with a larger diameter. Confirmatory prospective trials on patients with non-thrombosed false lumina are indicated.

## Data Availability

The authors declare that the data supporting the findings of this study are available within the article.

## Abbreviations

SMP: Shape memory polymer
CTA: Contrast-enhanced computed tomography
CIA: Common iliac artery
IMA: Inferior mesenteric artery
